# Epidemiological characteristics of and containment measures for COVID-19 in Busan, Korea

**DOI:** 10.4178/epih.e2020035

**Published:** 2020-06-01

**Authors:** Hyunjin Son, Hyojung Lee, Miyoung Lee, Youngduck Eun, Kyounghee Park, Seungjin Kim, Wonseo Park, Sora Kwon, Byoungseon Ahn, Dongkeun Kim, Changhoon Kim

**Affiliations:** 1 Busan Center for Infectious Disease Control and Prevention, Pusan National University Hospital, Busan, Korea; 2 Epidemic Intelligence Officer of Busan Metropolitan City, Busan, Korea; 3 National Institute for Mathematical Sciences, Daejeon, Korea; 4 Division of Health Policy, Busan Metropolitan City, Busan, Korea; 5 Department of Preventive Medicine, Pusan National University School of Medicine, Busan, Korea

**Keywords:** COVID-19, Containment measure, Reproduction number, Korea

## Abstract

**Objectives:**

To describe and evaluate epidemiological investigation results and containment measures implemented in Busan, where 108 cases were confirmed with coronavirus disease 2019 (COVID-19) between February 21, 2020 and March 24, 2020.

**Methods:**

Any individual who tested positive for COVID-19 was classified as a confirmed case. Measures were taken to identify the source of infection and trace and quarantine contacts. Serial intervals were estimated and the effective reproduction number was computed.

**Results:**

Of the total 18,303 COVID-19 tests performed between January 16, 2020 and March 24, 2020 in Busan, 108 yielded positive results (positive test rate, 0.6%). All confirmed cases were placed in isolation at hospitals. Of the 108 confirmed cases, 59 (54.6%) were female. The most common age group was 20-29 years with 37 cases (34.3%). Regarding symptoms at the time of diagnosis, cough (n=38, 35.2%) and fever (n=34, 31.5%) were most common; 12 cases (11.1%) were asymptomatic. The source of infection was identified in 99 cases (91.7%). A total of 3,223 contacts were identified and quarantined. Household contacts accounted for 196, and the household secondary attack rate was 8.2% (95% confidence interval [CI], 4.7 to 12.9). The mean serial interval was estimated to be 5.54 days (95% CI, 4.08 to 7.01). After February 26, (R_t_) remained below 1 in Busan.

**Conclusions:**

The early containment strategy implemented in Busan shows that control is possible if outbreaks are of limited scope. In preparation for future outbreaks, public health and healthcare systems should be re-examined and put in a ready state.

## INTRODUCTION

As of March 31, 2020, the global number of cases confirmed to have coronavirus disease 2019 (COVID-19) had surpassed 750,000, with the death toll at 36,000 [1]. Daily reports of numbers of newly confirmed cases have been above 50,000, showing that the disease is rapidly spreading worldwide. In Korea, a total of 9,786 cases and 165 deaths had been reported as of March 31, 2020 [2]. Between January 20, 2020, when the first case in the country was reported, and February 7, 2000, 24 cases were reported, and the source of infection was identified for each of these cases [3]. However, after February 28, 2020 when the #31 case (known as a member of a cult church called *Shincheonji*) was reported in Daegu city, reports of confirmed cases started increasing rapidly, and the number of cases without an identified source of infection rose [4]. On February 29, 2020, 909 cases, the highest daily reported number, were confirmed; after that, the daily case numbers decreased steadily. The total number of confirmed cases reported in Daegu was 6,684, constituting 68% of the total number of confirmed cases reported nationwide. As of the end of March, the daily case number remained around 100, whereas the number of imported cases consistently increased. Until March 31, 2020, a total of 518 imported cases from other countries had been reported. Of those, 282 (54.4%) arrived from Europe and 157 (30.3%) from the Americas [2].

Busan is a Korean metropolitan city, located at the southeastern coast of the Korean Peninsula, with a population of approximately 3,400,000. Busan is approximately 88 km away from Daegu, the city where 68% of the total confirmed cases in Korea were reported. The first case in Busan was reported on February 21, 2020 and as of March 31, 2020, a total of 119 cases had been reported. Here, we describe the epidemiological investigation results on the 108 confirmed cases reported in Busan as of March 24, 2020 and the containment measures that were implemented. Additionally, we evaluated the containment measures used in Busan by using important epidemiological indices (such as secondary infections within household and serial intervals) and the effective reproduction number (R_t_).

## MATERIALS AND METHODS

### Epidemiological investigation procedure and methods

All cases with positive results on COVID-19 diagnostic tests (real-time reverse transcription polymerase chain reaction; RTPCR) were classified as confirmed cases, irrespective of the presence or absence of symptoms, and required to report to a public health center. Public health centers and local autonomous governments were mandated to perform epidemiological investigations on all reported cases to identify the source of infection and find all contacts to place them in self-quarantine. A contact was defined as anyone who was in contact with a confirmed case from a day before the symptoms occurred, in a manner that offered the potential for transmission through respiratory droplets. The decision was made in consideration of mask wearing, contact duration, and environmental factors. For example, if both individuals were wearing masks and talked only briefly, the person was not classified as a contact. In contrast, anyone who had meal with a case at the same table was classified as a contact.

The investigation to identify the source of infection as well as contacts was initially based on patient statements. Immediately after a case was reported, the investigation began. It typically took about 1 hour to listen to a patient’s report. Because memories can be incomplete and patients sometimes hide information, objective data (including CCTV data, cell phone location information based on cell tower signals, and credit/debit card usage data) were additionally used to complete and confirm information.

Those classified as a contact and placed in self-quarantine were forbidden to leave home. Public workers at the corresponding local government called these individuals at least twice a day to check on symptom onset and body temperature. If symptomatic, these contacts were instructed to get tested. Contacts with high exposure levels, such as family members, were instructed to get tested regardless of the presence or absence of symptoms. The duration of self-quarantine was 14 days, counting from the day of the last contact with a confirmed case.

If a case belonged to a group/facility at high risk for transmission, like church members, hospital staff, or school teachers, a complete enumeration regarding symptoms was performed in the group/facility. Either all group/facility members or only those members who were symptomatic were tested.

### Serial intervals

Serial intervals were calculated using the data of infector-infectee pairs for whom the dates of symptom onset were reported. The distribution of serial intervals was assumed to follow a discretized gamma distribution. The discretized gamma distribution was defined as *f*(*t*)=*G*(*t*+0.5)-*G*(*t*-0.5), with *G*(*t*) representing a cumulative gamma distribution. The likelihood function was as follows:

$$L\left(\mu ,\sigma ,t\right)=\prod_{i=1}^{m}f(t_{i}^{infector}-t_{i}^{infectee};\;\mu ,\sigma ),$$

where *μ* and *σ* were the mean and the standard deviation, respectively, of the serial interval distribution. For a total of *m* infector-infectee pairs, $t_{i}^{infector}$ was the date of symptom onset in the *i*-th infector and $t_{i}^{infectee}$ was the date of symptom onset in the *i*-th infectee ($t=[t_{i}^{infector}-t_{i}^{infectee}]$). Serial intervals were estimated using maximum likelihood estimation (MLE). The 95% confidence interval (CI) of the mean and the standard deviation were obtained by computing the 2.5th and 97.5th percentiles based on parametric bootstrapping, using the Hessian matrix of the corresponding statistic estimates, employing MLE [5].

### Effective reproduction number

Based on the estimated serial interval, the renewal equation accounting for the imported cases from another city or foreign country was defined as follows [6].

$$c_{t}=R_{t}\sum_{\tau =0}^{t}(c_{t-\tau }+\alpha j{}_{t-\tau })f_{\tau },\;0\leq \alpha \leq 1$$

Here, *c_t_* indicated the number of symptomatic cases in the Busan area reported on day *t*(local cases), and *j_t_* indicated the number of symptomatic cases that imported from another city or a foreign country on the same day (imported cases). According to the parameter α, which represents the relative contribution of secondary transmission of imported cases, *R_t_* can be computed in consideration of two scenarios. First, new cases can be infected only by locally confirmed cases in the Busan area (i.e., α= 0). Second, if α≠0, imported cases from another city or foreign country can generate new cases. Finally, estimates of *R_t_* were computed over a time window τ to analyze the dynamics of COVID-19 [7-9]. The 95% CIs were obtained through parametric bootstrapping (1,000 replications) on the mean and standard deviation of the serial interval distribution.

### Ethics statement

This study was conducted by provincial government in accordance with the law in a situation where urgent measures are needed for public health.

## RESULTS

### Daily numbers of tests and rates of positive tests

**Figure 1.**
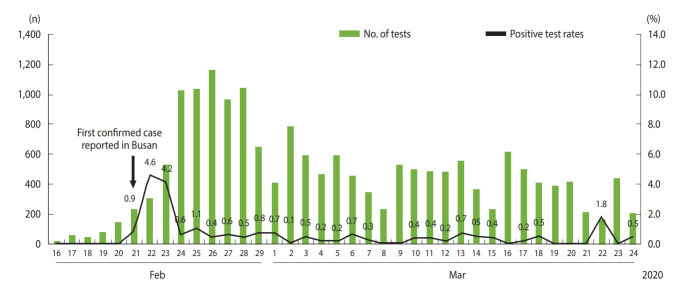
Daily numbers and positive test rates for coronavirus disease 2019 in Busan.

A total of 18,303 COVID-19 RT-PCR tests were performed in Busan between January 16, 2020 and March 24, 2020, with 108 yielding positive results (positive test rate, 0.6%). Daily test numbers and positive test rates are shown in Figure 1. The number of tests per day increased rapidly after February 21, 2020 when the first case in Busan was reported, and between February 24 and 28, 2020 approximately 1,000 tests were performed per day. The majority of these tests were conducted as part of epidemiological investigations, such as tests performed on contacts and tests for all group/facility members at high transmission risk.

### Characteristics of confirmed cases and the epidemic curve

All of the 108 confirmed cases were admitted to hospitals in isolation; 59 (54.6%) were female and 49 (45.4%) were male. The most common age group was 20-29 years (n = 37, 34.3%), followed by 50-59 years (n= 14, 13.0%) and 70 years or older (n= 13, 12.0%) (Table 1). Regarding symptoms at diagnosis, cough (n= 38, 35.2%) and fever (n= 34, 31.5%) were the most common, whereas 12 cases (11.1%) were asymptomatic. Sixty cases (59.8%) were diagnosed within 3 days after symptom onset. As of March 24, 2020, two cases had died, both aged 70 or older. One of these two had cardiovascular disease, whereas the other had no underlying diseases.

**Figure 2.**
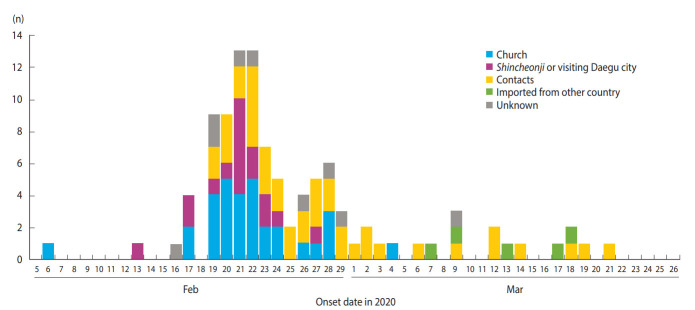
Epidemic curve of 102 confirmed cases according to source of infection.

Figure 2 shows the epidemic curve for 102 out of the 108 cases according to source of infection. Six cases were excluded, four who remained asymptomatic as of March 24, 2020 and two for whom the date of symptom onset was not clearly identified. Early in the study period, the number of cases rapidly increased due to the church cluster and imported cases from other cities. This was followed by a second peak centered around the contacts of first peak cases. Since March 7, 2020, cases infected from other countries and cases infected through contact with known confirmed cases have been sporadically reported.

### Relationship diagram of confirmed cases

**Figure 3.**
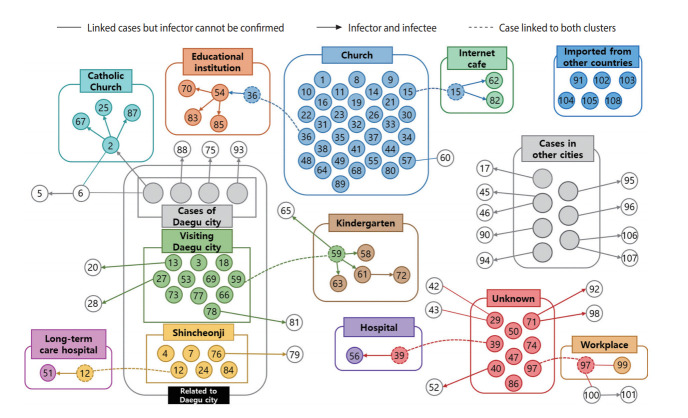
Relationship diagram of the 108 confirmed cases in Busan.

Of the 108 cases, the source of infection was identified in 99 (91.7%) and not identified in 9 (8.3%) cases (Figure 3). The largest cluster was the church cluster, comprising a total of 32 confirmed cases. Related to this cluster, two smaller clusters were found in an educational institution and in an Internet cafe. Additionally, three clusters in a kindergarten, a Catholic Church, and a long-term care hospital were identified among the confirmed cases linked to the Daegu outbreak.

### Contact tracing and quarantine

Based on contact tracing of the 108 cases, a total of 3,223 individuals were identified as contacts and quarantined. The number of contacts linked to a group/facility was the highest in the church, at 1,089, followed by the long-term care hospital (n= 296), a hospital (n= 159), the Catholic Church (n= 131), the kindergarten (n= 71), and the educational institution (n= 61). Tests were performed on all contacts of cases in the long-term care hospital, hospital, kindergarten, and educational institution, irrespective of the presence or absence of symptoms. Additionally, 128 contacts of the church cluster cases with high exposure levels were tested, regardless of the presence or absence of symptoms. In all other contacts, tests were only performed if symptoms developed during quarantine.

A total of 196 contacts were family members. Of those, 165 (84.2%) underwent COVID-19 real-time RT-PCR tests at least once before self-quarantine was over, and 16 tested positive. Thus, the household secondary attack rate (SAR) was 8.2% (95% CI 4.7 to 12.9).

### Serial intervals

Serial intervals were estimated based on the data from 28 pairs of 56 cases in whom the infector-infectee relationship was clearly identified (*i*= 1, 2, …, 28, *m*= 28). The mean serial interval was estimated to be 5.54 days (95% CI, 4.08 to 7.01) and the standard deviation 3.90 days (95% CI, 2.47 to 5.32).

### Effective reproduction number

**Figure 4.**
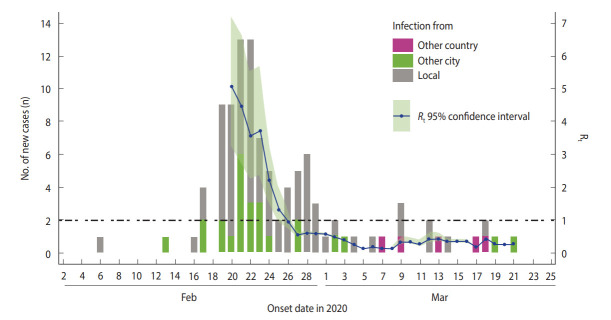
Epidemic curve and effective reproduction number (R_t_) in Busan.

The R_t_ was calculated based on the estimated serial interval. The time window τ was used at 5 days, which is a rounded-down value of the mean serial interval, as discussed above. The estimates of the R_t_ accounting for the imported cases, given α= 1 were shown in Figure 4. Initial Rt values were very high, but the values decreased rapidly. From February 26, 2020, the values remained below 1.

## DISCUSSION

When a new infectious disease breaks out while treatment and vaccines are not yet available, aggressive case finding and isolation as well as contact tracing and quarantine are representative control measures that aim for containment. These measures were successfully used in the past to control Severe Acute Respiratory Syndrome and Middle East Respiratory Syndrome (MERS) [10,11]. Particularly, to prevent onward transmission from the secondary cases, both case isolation and contact quarantine (i.e., placing all contacts of a case in quarantine for the maximum period of incubation) should be performed in combination, rather than case isolation alone [12]. COVID-19 modeling studies have also reported that the combination of the two containment measures had a great impact on the reduction of infection occurrence [13-15]. Non-pharmaceutical interventions (NPIs) by themselves cannot prevent the outbreak of a pandemic, and countries should also use a containment strategy, if possible [16].

In Busan, Korea, where relatively small COVID-19 outbreaks occurred without the large-scale superspreading events (SSEs) that were observed in Daegu [4,6], aggressive containment measures, investigating all confirmed cases to trace and quarantine contacts, could be conducted. Additionally, concentrated case management was made possible by admitting all cases to isolation units at hospitals designated for COVID-19. During the MERS outbreak in 2015, 154 secondary infections were traced back to just five out of the total of 186 cases reported in Korea, whereas no secondary infections occurred in 166 (89%) cases [17,18]. Since that experience with SSEs, the country has established aggressive management measures to limit visits to cases in hospitals and prevent nosocomial infections, which is believed to have played a large role in the current epidemic.

The basic reproduction number (R_0_), indicative of the transmission speed of an infectious disease, measures the average number of cases infected by a single case during the infectious period in a population of susceptible persons. In other words, a value of R0 over 1 means that the infection is spreading, while a value under 1 means that the outbreak will eventually die out. We computed the Rt to assess the containment strategy implemented in Busan. In Figure 4, R_t_ values were very high initially, when spreading from the church cluster was not yet detected. After February 21, 2020, when the first confirmed case had been reported, an aggressive containment strategy was implemented, and R_t_ values decreased to levels below 1 from February 26, 2020, suggesting that the outbreak was under control.

It is of note that the positive test rate was low, under 1%, because testing was aggressively performed from the beginning, and based on the 108 confirmed cases, a total of 3,223 contacts were quarantined. These measures reduced the time to isolate additionally confirmed cases as well as the number of additional contacts, which is believed to have contributed to the low level of Rt. Additionally, it can be speculated that the policy to test for COVID-19, if deemed necessary by a healthcare professional, implemented after testing capacities were increased from February 7, 2020 also contributed to reducing the delay between symptom onset and isolation and the rate of infection transmission prior to symptom onset and increasing the probability of a contact being traced. It can be concluded that the juxtaposition of proactive testing and an aggressive containment strategy of case isolation and contact quarantine enabled iterative tracing, i.e., repetitive as well as reverse-tracing instead of single-step tracing, that enhanced the outcome of the contact tracing measures [12].

The SAR in the Busan area was 8.2% (95% CI, 4.7 to 12.9), a level similar to the household SAR of 7.6% among the first 30 cases in Korea [19]. Although NPIs are implemented, increased contact rates within families do not seem to occur. The reason for the age group of 20–29-year-olds being the largest is supposedly that the outbreak occurred among individuals on church retreats for young adults and that a majority of those who visited Daegu were in their 20s, which influenced the age distribution of the confirmed cases during the first outbreak in the Busan area. The serial interval, i.e., the interval between symptom onset in an infectorinfectee pair, is an important epidemiological characteristic in understanding the speed of infection transmission. In this study, the estimated mean serial interval was 5.54 days, longer than the mean serial interval estimated for the first 24 cases in Korea, which was 4.6 days [3], but very close to the estimates based on 77 pairs of COVID-19 reported in China, which was 5.8 days [20].

So far, at least one cluster of asymptomatic cases has been reported [21], and follow-up studies on the transmissibility of asymptomatic COVID-19 should be conducted. Of the 108 cases in Busan, 12 (11.1%) were asymptomatic at diagnosis, and of those, 4 (3.7%) were confirmed to have remained asymptomatic as of March 24, 2020. This is in line with the findings at a call center in Seoul, where 8 (8.3%) out of 97 confirmed cases were asymptomatic at diagnosis and 4 (4.1%) remained asymptomatic even 14 days later [22]. Asymptomatic and presymptomatic transmissions hamper the effects of case isolation and contact quarantine and necessitate social distancing to be considered more aggressively. Thus, follow-up research should be conducted on rates of asymptomatic and presymptomatic transmission [14]. Ferguson et al. [16] found in a modeling study based on cases in the United Kingdom and United States that the number of cases could increase to more than eight times the current capacity of healthcare systems, even with the most optimal mitigation strategy. Their study clearly demonstrates what could happen if an aggressive containment strategy is not continuously maintained. The greatest challenges in sustaining containment strategies are public health infrastructure and concerns over civil liberties [23]. Furthermore, NPIs have not been applied or evaluated over long periods in real life. And no vaccine or effective antiviral drugs is likely soon and probably at least 12 months to 18 months away from substantial vaccine production [13,14]. Nonetheless, aggressive containment strategies should be maintained, because largescale outbreaks predicted to occur if strategies are unfastened will overwhelm available healthcare resources [24,25].

The situation in Busan and in Korea overall shows that control is possible when cases are isolated and contacts are quarantined through aggressive case tracing and wide-ranging testing. However, it is difficult to predict up to what incidence level such a containment strategy will work as the number of persons traveling from overseas increases and small-scale outbreaks persist, and what will happen if infection management interventions at the level of healthcare facilities do not work well.

The early containment measures described here show that control is implemented if an outbreak is of limited scope. This should be seen as having secured the time for preparation to develop sustainable containment strategies. Findings from epidemiological investigations of the early cases in Daegu suggest that if the number of cases increases and outbreaks continue, the likelihood for the measures currently implemented in Korea (case isolation, contact tracing, and contact quarantine) to work well is low, and infection management interventions at community hospitals may only provide limited prevention of nosocomial infections. Until now, in Busan, approximately 30 contacts per case were quarantined, in spite of the burden on the local health authorities. This level comes very close to the 36.1 suggested for an effective containment strategy in a modeling study conducted in the United Kingdom [26]. In order to achieve the highest possible capacity for the prevention of community spread, case and contact tracing studies should be performed, infrastructure should be created for high-level quarantine of contacts, additional hospitals should be equipped to cope with infection management, and contact screening capacities should be further improved.

If infected cases are not effectively isolated and infections spreading through hospitals are not blocked, containment strategies may not be as effective as they have been observed here. If hospitalbased SSEs are likely and if post-isolation infection prevention rates are not high, the effect of containment strategies will be limited. If such a situation occurs even though NPIs have been implemented and maintained for a long time, the drastic effect of outbreak control that we observed this time may not occur in the future [23,27].

Additionally, in preparation for an increase in the number of cases, planning should ensure that aside from the hospitals specialized for the treatment of COVID-19, mostly public hospitals, any institution with an internal capacity for infection management should be actively utilized to isolate and treat cases. As shown in Italy [28], the United States [24], and other countries where the pandemic is spreading, treating COVID-19 cases in available intensive care units (ICUs) only is largely insufficient. As of now, Korea has enough available ICUs at the regional level, but if the scope of outbreaks expands, healthcare demands will explode, in which case the capacity for intensive case treatment could be maximized only if within-hospital and between-hospital, and regional systems for intensive care treatment and incase management are in place [29-31]. Korea has never experienced the sensitive decision-making that frontline clinicians, hospital managers, and those working in public health and relevant fields will have to deal with now, in a race against the clock, in the event that a large number of severely ill cases are hospitalized; the country has also no experience with the ethical issues stemming from modifications of standard treatments in a time of crisis [32]. Currently, we expect that the number of new infections with COVID-19 will continue to remain at a very low level, but the course of outbreaks can change at any time. In preparation for a future outbreak, the country should proactively re-examine its public health and healthcare systems and ensure to be prepared.

## Figures and Tables

**Table 1.** Demographic characteristics and symptom distribution of all confirmed cases (n=108)

Characteristics	n (%)
Sex	
Female	59 (54.6)
Male	49 (45.4)
Age	
0-9	3 (2.8)
10-19	8 (7.4)
20-29	37 (34.3)
30-39	10 (9.3)
40-49	12 (11.1)
50-59	14 (13.0)
60-69	11 (10.2)
≥70	13 (12.0)
Symptoms at diagnosis	
Cough	38 (35.2)
Fever	34 (31.5)
Myalgia	20 (18.5)
Sore throat	20 (18.5)
Headache	14 (13.0)
Chill	12 (11.1)
Sputum	11 (10.2)
Rhinorrhea	6 (5.6)
Other symptoms	24 (22.2)
Asymptomatic	12 (11.1)

## SUPPLEMENTARY MATERIALS

Korean version is available at http://www.e-epih.org/.
