# Supplementary material for: Epidemiological characteristics of and containment measures for COVID-19 in Busan, Korea
Source: Epidemiol Health. 2020 Jun 1;42:e2020035. doi: 10.4178/epih.e2020035 (PMC7644939; doi:10.4178/epih.e2020035)
Supplement: Supplementary file 1 [file epih-42-e2020035-suppl.docx]

**제목**

**부산시에서 발생한 코로나바이러스감염증-19 환자의 역학적 특성과 관리 조치**

**Epidemiological characteristics of and containment measures for coronavirus disease 2019 in Busan, Korea**

Hyunjin Son^1,2^, Hyojung Lee^3^, Miyoung Lee^1,2^, Youngduck Eun^1,2^, Kyounghee Park^1^,

Seungjin Kim^1^, Wonseo Park^1^, Sora Kwon^1^, Byoungseon Ahn^4^, Dongkeun Kim^2,4^,

Changhoon Kim^1,5^

^1^Busan Center for Infectious Disease Control and Prevention, Pusan National University Hospital, Busan, Korea; ^2^Epidemic Intelligence Officer of Busan Metropolitan City, Busan, Korea; ^3^National Institute for Mathematical Sciences, Daejeon, Korea; ^4^Division of Health Policy, Busan Metropolitan City, Busan, Korea; ^5^Department of Preventive Medicine, Pusan National University School of Medicine, Busan, Korea

**초록**

Objectives: 2월 21일부터 3월 24일까지 부산시에서 보고된 108명의 코로나바이러스감염증-19 (코로나19) 확진 환자의 역학 조사 결과와 관리 조치를 기술하고 평가하고자 하였다.

Methods: 코로나19 진단 검사(real-time RT-PCR) 에서 양성인 모든 경우 증상 여부에 상관없이 확진 환자로 분류하였다. 모든 확진 사례에 대해 감염원을 밝히고 접촉자를 찾아 격리하는 조치를 시행하였다. 이산화된 감마분포와 최대우도추정법을 이용하여 연속감염기간을 추정하였고, 타지역 또는 타국가로부터의 유입을 고려한 실제재생산수를 계산하였다.

Results: 부산에서 1월 16일부터 3월 24일까지 18,303 건의 코로나19 검사가 시행되었으며, 양성 건수는 108 건으로 양성률은 0.6%였다. 108명의 확진 환자는 모두 병원에 격리 입원하였다. 여성은 59명(54.6%) 남성은 49명(45.4%)이었고, 20대가 37명(34.3%)으로 가장 많았다. 진단 당시 증상으로는 기침이 38명(35.2%), 발열이 34명(31.5%)으로 가장 많았으며 무증상인 상태에서 진단된 경우는 12명(11.1%)이었다. 초기에는 A 교회 클러스터와 다른 지역에서 유입된 사례로 인해 급격한 환자 증가가 있었고 이후 접촉자를 중심으로 2차 유행을 보였다. 99명(91.7%)은 감염원을 밝힐 수 있었으며 9명(8.3%)은 감염원을 알 수 없었다. 108명의 환자에 대한 접촉자 추적 결과 접촉자로 확인되어 격리된 사람은 총 3,223명 이었다. 가족 접촉자는 196명이었고 가족 내 2차 전파율 (Secondary attack rate, SAR)은 8.2%(95% 신뢰구간 4.7% - 12.9%)였다. 연속감염기간은 평균 5.54일(95% 신뢰구간 4.08일 – 7.01일), 표준편차 3.90일(95% 신뢰구간 2.47일 – 5.32일)로 추정되었다. 2월 26일 이후 R_t_가 1보다 낮은 값을 보여 전파가 통제되고 있음을 알 수 있었다.

Conclusions: 부산에서 초기 containment 전략을 시행한 결과 제한된 발생 규모인 경우 통제가 가능하다는 점을 확인하였다. 이후 발생할 유행에 대비해 적극적으로 공중보건 및 보건의료 체계를 정비하고 준비해야 할 것으로 생각된다.

**중심 단어**

COVID-19, Containment measure, Effective reproduction number, Korea

**서론**

3월 31일 현재 전세계 코로나바이러스감염증-19 (코로나19) 확진 환자는 75만명, 사망자는 3만 6천명을 넘어섰다 [1]. 매일 보고되는 환자수도 5만명 이상으로 매우 빠른 속도로 전세계에서 확산 중이다. 한국에서는 3월 31일까지 9,786명의 환자와 165명의 사망자가 보고되었다 [2]. 1월 20일 첫 환자가 보고된 이후 2월 7일까지 24명의 환자가 보고되었고 모두 감염경로가 명확하였다 [3]. 그러나 2월 18일 신천지 교인으로 알려진 한국의 31번째 환자가 대구에서 보고된 이후 환자 보고가 급격히 증가하기 시작하면서 감염경로를 알 수 없는 사례가 증가하였다 [4]. 2월 29일 909명의 환자가 보고되어 일일 최대 환자수를 기록한 이후 꾸준히 감소하여 3월 15일 76명으로 일일 보고 환자 수가 처음으로 100명 이하로 감소하였다. 한국의 17개 광역자치단체 중 대구시에서 보고된 환자는 6,684명으로 68%를 차지하고 있다. 3월 말 현재 하루 100명 내외의 환자가 지속적으로 보고되고 있으며 해외유입 사례가 지속적으로 증가하고 있는 상황이다. 3월 31일까지 518명의 해외 유입 사례가 보고되었으며 유럽이 282명(54.4%) 아메리카가 157명(30.3%)을 차지하고 있다 [2].

부산은 한국의 남동쪽 해안에 위치한 대도시로 인구는 약 340만명이다. 한국에서 보고된 환자의 68%를 차지하고 있는 대구시와 직선거리로 약 88Km 떨어진 곳에 위치하고 있다. 부산에서는 2월 21일 첫 환자가 보고되었고 3월 31일 현재까지 총 119명의 환자가 보고되었다. 이 연구에서는 3월 24일까지 부산에서 보고된 108명의 확진환자 역학조사 결과와 관리 조치를 상세히 기술하였다. 또한 가족 내 2차 발병률, 연속감염기간(serial interval) 등 주요한 역학적 지표를 산출하였으며 실제재생산수(effective reproduction number, $R_{t}$)를 산출하여 부산의 관리 조치에 대해 평가하고자 하였다.

**연구방법**

1. 역학조사 절차와 방법

코로나19 진단 검사(real-time RT-PCR) 에서 양성인 모든 경우 증상 여부에 상관없이 확진 환자로 분류되며 반드시 보건소로 신고해야 한다. 보건소와 광역자치단체는 신고된 모든 사례에 대해 역학조사를 실시하여 감염원을 밝히고 접촉자를 찾아 자가격리 조치를 시행한다. 접촉자는 증상 발생 1일 전부터 비말 전파가 가능한 접촉을 한 모든 사람으로 정의하고 있으며, 마스크 착용 여부, 접촉 시간, 환경 요인 등을 고려하여 판단한다. 예를 들어 서로 마스크를 착용한 상태로 신체적 접촉 없이 짧은 대화를 나눈 경우는 접촉자로 분류되지 않으며, 같은 테이블에서 식사를 같이 한 경우는 모두 접촉자로 분류한다.

감염원을 찾고 접촉자를 찾기 위한 조사는 기본적으로 환자의 진술을 기반으로 하고 있다. 환자가 신고되면 즉시 조사가 시작되며 환자의 진술을 듣는 데 대개 한 시간 이상의 시간이 소요된다. 환자의 기억이 불완전할 수 있고 때로는 숨기는 정보도 있기 때문에 CCTV, 기지국 기반 휴대전화 위치정보, 카드사용 내역 등 객관적 자료를 이용하여 조사의 완결성을 높이고 있다.

접촉자로 분류되어 자가격리를 하는 경우 외출은 금지되며 해당 기초자치단체 공무원이 하루에 2번 이상 전화로 증상유무와 체온을 확인하고 증상이 있는 경우 검사를 받도록 하고 있다. 가족 접촉자와 같이 노출의 강도가 높은 경우 증상 여부와 관계없이 검사를 받도록 하고 있다. 자가격리 기간은 확진 환자와의 마지막 접촉일로부터 14일이 되는 날까지이다.

확진 환자가 교회나 병원, 학교와 같이 전파 위험이 높은 집단 시설에 속한 경우 해당 집단 시설 전수를 대상으로 증상 여부를 조사하여 증상이 있는 경우 검사를 하거나 혹은 증상 여부에 관계없이 전수 검사를 하기도 한다.

1. 연속감염기간 추정

감염자(Infector)와 피감염자(Infectee)의 증상발생일이 보고된 쌍(pair)의 데이터로부터 연속감염기간을 계산하였다. 이산화된 감마분포(discretized gamma distribution)를 연속감염기간의 분포로 가정하였다. 즉, 누적감마분포를 $G(t)$라고 할 때, 이산화된 감마분포는$f\left( t \right)=G\left( t+0.5 \right)-G(t-0.5)$로 정의했으며, 우도추정함수(likelihood function)는 다음과 같이 표기할 수 있다.

$$L\left( \mu, \sigma;\boldsymbol{t} \right)=\prod_{i=1}^{m} f\left( t_{i}^{infector}-t_{i}^{infectee};\mu, \sigma\right),$$

$\mu, \sigma$는 각각 연속감염기간 분포의 평균, 표준편차를 의미한다. 감염자-피감염자 총 *m* 쌍에서, $t_{i}^{infector}$는 i번째 감염자의 증상발현일, $t_{i}^{infectee}$는 *i*번째 피감염자의 증상발생일을 나타낸다 ($\boldsymbol{t}=\left\{ t^{infector},t^{infectee} \right\}$). 최대우도추정법(maximum likelihood estimation, MLE)을 이용하여 추정하였다. 95% 신뢰구간은 연속감염기간(serial interval)의 최대우도추정법(MLE)을 통해 예측된 평균, 표준편차에 대한 Hessian matrix를 활용하여, 모수적 붓스트랩(Parametric bootstrap)을 통해 2.5분위수(percentile), 97.5분위수(percentile)를 계산하여 구하였다 [5].

1. 실제재생산수 계산

위에서 추정한 연속감염기간을 활용하고, 부산지역으로의 외부유입(Imported case)을 고려한 수리적 모델을 다음과 같이 정의하였다 [6].

$$c_{t}=R_{t}\sum_{\tau=0}^{t} \left( c_{t-\tau}+\alpha j_{t-\tau} \right)f_{\tau}, 0\leq\alpha\leq1.$$

여기서, $c_{t}$는 *t*일의 부산지역에서 발생한 증상발현 환자수 (Local case), $j_{t}$는 타지역 또는 타국가로부터 유입된 증상발현 환자수 (Imported case)를 나타낸다. $R_{t}$는 두가지 가정에서 계산되었다. 첫째, 부산지역에서 발생한 환자에 의한 새로운 감염환자만을 고려한다 ($\alpha=0).$ 둘째, $\alpha\neq0$일 때는 타지역 또는 타국가로부터 유입된 환자를 포함하여 새로운 감염환자 발생을 고려한다. Time window를 고려하여, time window 기간 $\tau$ 동안은 $R_{t}$가 상수라는 가정을 토대로, $R_{t}$를 계산하여 증감의 패턴을 분석하였다 [7–9]. 95% 신뢰구간은 연속감염기간의 분포의 예측치인 평균, 표준편차에 대한 1000번의 모수적 붓스트랩 방법을 통해 계산하였다.

**결과**

1. 일별 검사 건수와 양성률

부산에서 1월 16일부터 3월 24일까지 18,303 건의 코로나19 PCR 검사가 시행되었으며 그 중 총 양성 건수는 108 건으로 양성률은 0.6%였다. 일별 검사 건수와 양성률 추이는 Figure 1과 같다. 부산에서 첫 환자가 보고된 2월 21일이후 검사 건수가 급격히 증가하여 2월 24일부터 28일까지 하루 1,000건 내외의 검사가 시행되었으며, 이는 접촉자 검사와 집단 시설 전수 조사 등 역학 조사로 수행된 검사가 다수를 차지하고 있다.

1. 확진 환자 특성과 유행 곡선

부산에서 3월 24일까지 진단된 108명의 확진 환자는 모두 병원에 격리 입원하였다. 이 중 여성은 59명(54.6%) 남성은 49명(45.4%)이었다. 20대가 37명(34.3%)으로 가장 많았으며 50대가 14명(13.0%)이었고 70대 이상은 13명(12.0%)이었다 (Table 1). 진단 당시 증상으로는 기침이 38명(35.2%), 발열이 34명(31.5%)으로 가장 많았으며 무증상인 상태에서 진단된 경우는 12명(11.1%)이었다. 60명(59.8%)은 증상 발생 후 3일 이내에 진단되었다. 3월 24일 현재 2명이 사망하였는데 모두 70대 이상이었으며, 한 명은 심혈관 질환이 있던 환자였고 한 명은 기저질환이 없었다.

108명의 환자 중 3월 24일 현재까지 무증상인 4명과 증상발생일이 불분명한 2명을 제외한 102명의 감염경로 별 유행곡선은 Figure 2와 같다. 유행 초기에는 A 교회 클러스터와 다른 지역에서 유입된 사례로 인해 급격한 환자 증가가 있었고 이후 접촉자를 중심으로 2차 유행을 보였다. 3월 7일 이후에는 해외 유입 사례와 접촉자에서 산발적으로 환자가 보고되고 있다.

1. 확진 환자 관계도

108명의 환자 중 99명(91.7%)은 감염원을 밝힐 수 있었으며 9명(8.3%)은 감염원을 알 수 없었다 (Figure 3). 가장 큰 클러스터는 A 교회에서 발생하였으며 총 32명의 환자가 확진되었고 이 클러스터와 관련되어 학원과 PC 방에서 작은 클러스터가 발생하였다. 대구시와 관련된 확진 사례에서 3개의 클러스터가 유치원, 성당 B, 요양병원에서 발생하였다.

1. 접촉자 추적과 격리

108명의 환자에 대한 접촉자 추적 결과 접촉자로 확인되어 격리된 사람은 총 3,223명 이었다. 집단시설과 관련된 접촉자는 A 교회에서 1,089명으로 가장 많았으며 요양병원 296명, 병원 159명, B 성당 131명, 유치원 71명, 학원 61명 순이었다. 요양병원, 병원, 유치원, 학원의 경우 접촉자 전수를 대상으로 증상 여부에 관계없이 검사를 시행하였으며, A 교회 접촉자 중 접촉의 강도가 높았던 128명을 대상으로 증상 여부에 관계없이 검사를 시행하였다. 그 외의 경우에는 격리 기간 중 증상이 발생하는 경우 검사를 시행하였다.

가족 접촉자는 196명이었는데, 이 중 165명(84.2%)은 격리 해제 전 적어도 한번 이상 PCR 검사를 받았으며 이 중 16명이 양성 결과를 보여 가족 내 2차 전파율 (Secondary attack rate, SAR)은 8.2%(95% 신뢰구간 4.7% - 12.9%)였다.

1. 연속감염기간 (serial interval)

감염자(Infector)와 피감염자(Infectee)의 관계가 분명한 56명(28쌍)의 데이터로부터 연속감염기간을 계산하였다 (*i*=1, 2, …, 28, *m*=28). 연속감염기간은 평균 5.54일(95% 신뢰구간 4.08일 – 7.01일), 표준편차 3.90일(95% 신뢰구간 2.47일 – 5.32일)로 추정되었다.

1. 실제재생산수 (effective reproduction number, R_t_)

추정된 연속감염기간을 이용하여 실제재생산수($R_{t}$)를 계산하였다. 외부유입을 고려한 ($\alpha=1)$ 실제감염재생산수($R_{t}$)와 95% 신뢰구간을 Figure 4에 나타내었다. Time window 기간 $\tau$ 는 위에서 계산한 평균연속감염기간에서 소수점 이하를 버린 5일을 사용하였다. 초기 $R_{t}$ 는 매우 높았으나 점차 감소하여 2월 26일 이후의 $R_{t}$는 1보다 낮아져 지속적으로 유지되고 있음을 확인할 수 있었다.

**토론**

새로운 감염병이 발생하여 치료제와 예방백신이 없는 경우, 적극적 환자 발견과 격리(isolation), 접촉자 추적조사 및 격리(quarantine)는 봉쇄(containment)를 목적으로 사용하는 대표적인 감염병 관리초치이며 [Severe Acute Respiratory Syndrome(](https://www.who.int/csr/sars/en/)SARS), Middle East Respiratory Syndrome(MERS)를 성공적으로 통제한 수단이었다 [10,11]. 특히, 추가 감염을 차단하기 위해서는 환자 격리(case isolation) 단독 조치보다는 환자와 접촉한 모든 사람을 최장 잠복기까지 격리하는 조치(quarantine contacts)가 함께 시행되어야 한다 [12]. 코로나19에 대한 모델링 연구에서도 두 가지 조치의 조합이 발생 경감에 미치는 영향이 크다고 알려져 있다 [13–15]. 비약물적 조치(non-pharmaceutical intervention, NPI)는 환자발견과 격리, 접촉자 추적조사와 함께 병행하여 사용하거나, 단독으로 사용할 경우 유행규모를 줄일 수 있는 공중보건학적 조치이다. 그러나 NPI 단독으로는 이후의 유행발생을 막을 수 없으며, 시행할 수 있는 국가의 경우 억제전략을 활용하는 것이 필요하다 [16].

대구 지역과 같은 대규모의 수퍼전파 사건(superspreading events, SSE) 없이 [4,6] 비교적 소규모의 유행이 발생한 부산의 경우 적극적인 봉쇄 전략에 따라 확진 환자 전수에 대한 접촉자 추적과 격리가 가능했다. 또한, 전담병원을 지정하여 모든 환자를 입원격리 함으로써 집중적인 환자관리를 시행할 수 있었다. 2015년 메르스 유행 시 한국에서 보고된 환자 총 186 명 중 166 명 (89 %)은 이차 전파 사례가 없었지만 5 명의 환자가 154 건의 2 차 사례를 발생시킨 SSE를 경험한 후 [17,18] 병원 면회 제한, 병원감염 차단을 위한 적극적인 관리조치를 도입한 것이 큰 역할을 한 것으로 생각된다. 일반 의료기관도 선별절차를 도입하여 감염주의 조치 수준을 높여 의료기관을 통한 유행의 발생도 억제할 수 있었다. 부산의 일부 병원에서 발생한 사례의 경우에도 보건의료종사자와 지역사회의 접촉제한 조치를 높은 수준으로 유지하여 감염이 확산되는 정도를 줄일 수 있었다.

기초감염재생산수(Basic reproduction number, $R_{0}$)는 감염병의 전파 속도를 나타내는 수치로써, 감염기간 동안 모두 감수성 있는 사람으로 구성된 인구 집단에서 감염환자 1명이 평균적으로 감염시키는 사례 수를 나타낸다 즉, $R_{0}$가 1보다 클 때, 감염이 확산될 수 있음을 의미하며 1보다 적을 때 유행이 통제되고 있음을 의미한다. 관리 조치가 취해질 때는 실제재생산수($R_{t}$)를 계산함으로써, 정책의 효과를 고려한 유행의 확산여부를 판단할 수 있다. 초기에 부산에서는 A교회를 중심으로 한 전파가 인지되지 못한 상황에서 매우 높은 실제재생산수를 나타내었고 첫 환자가 보고된 2월 21일 이후의 적극적 억제전략을 통해 2월 26일 이후 실제재생산수가 1이하로 나타나 유행이 통제되고 있음을 보여주었다.

초기에 많은 검사 이루어져서 1% 이하의 낮은 양성률을 나타낸 것과 108명의 환자에 대해 3,223명의 접촉자 격리조치를 시행한 것은 주목해야 할 지점이다. 이는 추가로 확인되는 환자의 격리로 이어지는 시간과 추가 접촉자를 감소시키는 효과를 가져왔다. 따라서 실제재생산수(R_t_)를 낮게 유지 하는데 기여하였을 것으로 생각된다. 이와 함께 2월 7일 가용한 검사 역량이 증가한 후 의료인이 필요하다고 판단할 경우 검사가 가능하도록 만든 정책은 증상 발생에서 격리까지의 지연(delay), 접촉이 추적 될 확률, 증상 발병 이전에 발생한 감염전파 비율을 감소시키는 데 기여하였을 것이다. 적극적인 봉쇄전략으로 시행된 환자와 접촉자 격리조치와 적극적인 검사가 병행되어 단일 단계 추적 (single-step tracing)이 아닌 추적-역추적을 반복하며 시행하는 반복적 추적조사 (iterative tracing)로 이어져, 추적 조사의 성과를 높였을 것으로 판단된다 [12].

한국 초기 환자 30명 대상 household SAR 7.56% [19]과 비교하였을 때 부산지역의 SAR은 8.2%(95% 신뢰구간 4.7% - 12.9%)로 유사한 수준이었다. NPI가 시행될 경우 가족 내 접촉률이 증가하는 것과 같은 효과는 나타나지 않은 것으로 생각된다. 연령분포 20대가 가장 많은 이유는 A 교회 청년부 수련회 참석자 중심 유행과 대구 방문자가 주로 20대인 점이 초기 부산지역 유행의 연령별 발생자 분포에 영향을 준 것으로 생각된다. 연속감염기간(serial interval)은 환자(Infector)의 증상발현 후 다음 감염자(Infectee)의 증상발현까지의 기간을 나타내며, 전파 속도를 이해하기 위한 중요한 역학적 특성이다. 이 연구에서 계산한 연속감염기간은 5.54일 이었는데, 국내 초기 환자 24명 자료를 이용하여 계산한 4.6일 [3] 보다는 길었으나 중국에서 발표된 77쌍의 공개적으로 이용가능 한 자료로 계산된 5.8일과 매우 유사하였다 [20].

그간 적어도 하나이상의 클러스터가 보고된 [21] 무증상감염의 전파가능성에 대해서도 후속 연구가 필요할 것으로 생각된다. 부산에서 확진된 108명 중 무증상으로 발견된 12명(11.1%) 중 24일까지 무증상 사례가 4명(3.7%)이 확인되었다. 이는 서울의 콜센터에서 발생한 유행에서 97명의 확진 환자 중 무증상 상태로 발견된 환자가 8명(8.3%)이고 그 중 4명(4.1%)은 14일 이후까지 무증상이었다는 결과와 유사하다 [22]. 무증상 감염과 무증상기 전파는 격리와 접촉자 검역의 효과를 낮출 수 있고 사회적 거리두기를 통한 감염차단을 더 적극적으로 고려해야 하는 만큼 무증상 감염율과 무증상기 감염력에 대한 후속연구가 필요하다 [14]. Ferguson 등이 영국과 미국을 대상으로 한 모델링에서 최적의 완화 정책으로도 보건의료 체계 수용력의 8배가 넘는 환자가 발생한다는 결과를 보여주었다 [16]. 이는 적극적인 억제정책을 지속적으로 유지하지 않았을 경우의 발생할 수 있는 상황을 잘 보여주고 있다. 공중보건인프라, 인권에 대한 침해 우려 등 봉쇄전략을 적극적으로 도입하기 어려운 상황 [23]과 백신과 효과적인 치료제가 개발될 때까지 12~18개월 간 장기간에 걸친 NPI 조치 시행의 경험과 사례가 없음 [13,14]은 지속적인 억제정책 시행에 있어 큰 도전이다. 그럼에도 불구하고 이를 완화할 경우 예상되는 대규모 유행은 가용한 의료자원을 압도할 것으로 예상되므로 [24,25] 가능한 강력한 억제 정책을 유지할 필요가 있다.

부산과 한국의 상황은 적극적인 추적조사와 광범위한 검사를 시행하여 환자 격리, 접촉자 격리 조치가 가능한 상황에서의 통제가능성을 보여준다. 그러나 국외 입국자가 증가하고 소규모의 유행이 지속되는 상황에서 억제전략이 어느 수준의 발생수준까지 작동할 수 있을지, 의료기관의 감염관리조치가 잘 작동하지 않을 경우에 어떤 상황에 처할 수 있을지 예측하기는 어렵다.

초기 containment 전략을 시행한 결과 제한된 발생 규모인 경우 통제가 가능하다는 점을 확인하였고, 이후 지속 가능한 억제정책 개발을 위한 준비기간을 확보한 것으로 판단해야 할 것이다. 유행가능성이 계속되는 동안 초기 유행 대응에서 병목들을 파악하고 보완해 나가야 할 것이다. 초기 대구에서 발생한 사례의 역학조사과정에서 확인된 사실은 환자사례가 증가하고 이들과 관련된 유행이 지속될 때 한국사회가 갖추고 있는 환자격리, 접촉자 격리, 추적조사가 충분히 작동하지 않을 가능성이 높다는 것이다. 그리고 지역사회 병원의 감염병 관리 조치가 병원 감염을 차단하는데 한계가 있을 수 있다는 것이다. 지금까지는 보건당국의 부담에도 불구하고 부산에서 1사례 당 약 30명의 접촉자(108명에 대해 3,223명)를 격리하였다. 이는 영국의 모델링 연구에서 제시한 효과적인 봉쇄전략을 위해 제안한 36.1명에 거의 근접하는 수준이다 [26]. 최대한 지역사회 감염전파차단을 위한 역량을 갖추기 위해 사례조사, 접촉자추적조사, 접촉자 격리수준을 높게 유지할 수 있는 인프라 확보와 높은 수준의 감염관리가 가능한 병원의 추가 확보, 접촉자의 검역수준을 높게 유지할 수 있는 역량의 보완이 이루어져야 할 것으로 생각된다.

감염자의 효과적인 격리와 의료기관을 통한 감염확산 차단이 가능하지 못할 경우 봉쇄전략이 지금까지의 수준으로 효과적이지 않을 가능성도 있다. 의료기관을 기반으로 한 SSE 가능성과 함께 환자 격리 후 감염 차단율이 높지 않을 경우 봉쇄정책의 영향은 제한적일 가능성이 높다. NPI를 오랜 기간 시행하였음에도 이런 상황이 발생한다면 이후에 발생할 유행에는 이번과 같은 극적인 유행차단 효과가 나타나지 않을 수도 있다 [23,27].

이와 함께 환자 발생 증가에 대비하여 공공병원 위주의 전담병원에 더해 감염병 관리 기관들 중 내부에 감염병 관리 역량이 갖추어진 병원에서도 적극적으로 환자를 격리하고 치료할 수 있도록 계획을 수립하는 것이 필요하다. 유행이 확산되고 있는 이탈리아 [28], 미국 [24] 등의 사례에서 알려진 바와 같이 가용한 중환자실 만으로 코로나19 환자를 진료하기에 매우 부족한 것으로 나타나고 있다. 아직은 각 지역별로 가용한 중환자실이 부족한 상황은 아니지만 유행의 규모가 커질 경우 의료수요 폭증에 따른 병원 별, 병원 간, 지역내 중환자치료와 입원환자관리체계를 구축 [29–31]해야만 중환자치료 역량을 최대한 활용할 수 있을 것이다. 이들 과정에서 대규모 중환자 발생 시 일선 임상의사, 병원관리자, 공중보건 또는 관련 업무와 관련된 이들이 감당해야 할 촌각을 다투는 민감한 의사결정과 위기 시 표준 진료의 변화와 관련된  윤리적 논의들 [32] 또한 한국 사회에서 해본 경험이 없다. 현재 한국의 상황은 모두가 이대로 매우 낮은 수준의 환자 발생이 지속되기만을 원하고 있지만 이후의 과정이 어떻게 변화할 지 그 누구도 예상할 수 없다. 이후 발생할 유행에 대비해 적극적으로 공중보건 및 보건의료 체계를 정비하고 준비해야 할 것으로 생각된다.

**Acknowledgements**

This project was supported by Government-wide R&D Fund Project for Infectious Disease Research (GFID), Republic of Korea (Grant No. HG18C0088).

부산광역시와 16개 구군 보건소, 환자치료에 힘쓰시는 의료진들을 비롯해 코로나바이러스감염증-19 대응을 위해 곳곳에서 최선을 다하고 계신 모든 분들께 깊은 감사를 드립니다.

**Conflict of Interest**

The authors have no conflicts of interest to declare for this study.

**References**

1. World Health Organization. *Coronavirus Disease 2019 Situation Report-71*.; 2020. https://www.who.int/dg/speeches/detail/who-director-general-. Accessed April 1, 2020.

2. The updates on COVID-19 in Korea as of 31 March. *Press Release*. https://www.cdc.go.kr/board/board.es?mid=&bid=0030. Accessed April 1, 2020.

3. Ki M. Epidemiologic characteristics of early cases with 2019 novel coronavirus (2019-nCoV) disease in Republic of Korea. *Epidemiol Health*. 2020;42. doi:10.4178/epih.e2020007

4. Coronavirus Disease-19: The First 7,755 Cases in the Republic of Korea. *Osong Public Heal Res Perspect*. 2020;11(2):85-90. doi:10.24171/j.phrp.2020.11.2.05

5. Akhmetzhanov AR, Lee H, Jung S mok, Kayano T, Yuan B, Nishiura H. Analyzing and forecasting the Ebola incidence in North Kivu, the Democratic Republic of the Congo from 2018–19 in real time. *Epidemics*. 2019;27:123-131. doi:10.1016/j.epidem.2019.05.002

6. Shim E, Tariq A, Choi W, Lee Y, Chowell G. Transmission potential and severity of COVID-19 in South Korea. *Int J Infect Dis*. March 2020. doi:10.1016/j.ijid.2020.03.031

7. Thompson RN, Stockwin JE, van Gaalen RD, et al. Improved inference of time-varying reproduction numbers during infectious disease outbreaks. *Epidemics*. 2019. doi:10.1016/j.epidem.2019.100356

8. Cori A, Ferguson NM, Fraser C, Cauchemez S. A New Framework and Software to Estimate Time-Varying Reproduction Numbers During Epidemics. *Am J Epidemiol*. 2013;178(9):1505-1512. doi:10.1093/aje/kwt133

9. Dalziel BD, Lau MSY, Tiffany A, et al. Unreported cases in the 2014-2016 Ebola epidemic: Spatiotemporal variation, and implications for estimating transmission. *PLoS Negl Trop Dis*. 2018;12(1). doi:10.1371/journal.pntd.0006161

10. Tognotti E. Lessons from the History of Quarantine, from Plague to Influenza A. *Emerg Infect Dis*. 2013;19(2):254-259. doi:10.3201/eid1902.120312

11. GENSINI G. The concept of quarantine in history: from plague to SARS. *J Infect*. 2004;49(4):257-261. doi:10.1016/j.jinf.2004.03.002

12. Klinkenberg D, Fraser C, Heesterbeek H. The effectiveness of contact tracing in emerging epidemics. *PLoS One*. 2006. doi:10.1371/journal.pone.0000012

13. Hellewell J, Abbott S, Gimma A, et al. Feasibility of controlling COVID-19 outbreaks by isolation of cases and contacts. *Lancet Glob Heal*. 2020;8(4):e488-e496. doi:10.1016/S2214-109X(20)30074-7

14. Anderson RM, Heesterbeek H, Klinkenberg D, Hollingsworth TD. How will country-based mitigation measures influence the course of the COVID-19 epidemic? *Lancet*. 2020;395(10228):931-934. doi:10.1016/S0140-6736(20)30567-5

15. Kucharski AJ, Russell TW, Diamond C, et al. Early dynamics of transmission and control of COVID-19: a mathematical modelling study. *Lancet Infect Dis*. March 2020. doi:10.1016/S1473-3099(20)30144-4

16. Ferguson NM, Laydon D, Nedjati-Gilani G, et al. Impact of non-pharmaceutical interventions (NPIs) to reduce COVID-19 mortality and healthcare demand. *Imp Coll COVID-19 Response Team*. 2020. doi:10.25561/77482

17. Kim SW, Park JW, Jung HD, et al. Risk factors for transmission of Middle East respiratory syndrome coronavirus infection during the 2015 outbreak in South Korea. *Clin Infect Dis*. 2017. doi:10.1093/cid/ciw768

18. Chun BC. Understanding and Modeling the Super-spreading Events of the Middle East Respiratory Syndrome Outbreak in Korea. *Infect Chemother*. 2016;48(2):147. doi:10.3947/ic.2016.48.2.147

19. Coronavirus Disease-19: Summary of 2,370 Contact Investigations of the First 30 Cases in the Republic of Korea. *Osong Public Heal Res Perspect*. 2020;11(2):81-84. doi:10.24171/j.phrp.2020.11.2.04

20. He X, Lau EHY, Wu P, et al. Temporal dynamics in viral shedding and transmissibility of COVID-19. *Nat Med*. 2020;26(5):672-675. doi:10.1038/s41591-020-0869-5

21. Yu P, Zhu J, Zhang Z, Han Y. A Familial Cluster of Infection Associated With the 2019 Novel Coronavirus Indicating Possible Person-to-Person Transmission During the Incubation Period. *J Infect Dis*. February 2020. doi:10.1093/infdis/jiaa077

22. Park SY, Kim Y-M, Yi S, et al. Coronavirus Disease Outbreak in Call Center, South Korea. *Emerg Infect Dis*. 2020;26(8). doi:10.3201/eid2608.201274

23. Lloyd-Smith JO, Galvani AP, Getz WM. Curtailing transmission of severe acute respiratory syndrome within a community and its hospital. *Proc R Soc B Biol Sci*. 2003. doi:10.1098/rspb.2003.2481

24. Ranney ML, Griffeth V, Jha AK. Critical Supply Shortages — The Need for Ventilators and Personal Protective Equipment during the Covid-19 Pandemic. *N Engl J Med*. 2020. doi:10.1056/NEJMp2006141

25. Hick JL, Biddinger PD. Novel Coronavirus and Old Lessons — Preparing the Health System for the Pandemic. *N Engl J Med*. March 2020:NEJMp2005118. doi:10.1056/NEJMp2005118

26. Keeling MJ, Hollingsworth TD, Read JM. The Efficacy of Contact Tracing for the Containment of the 2019 Novel Coronavirus (COVID-19). *medRxiv*. 2020. doi:10.1101/2020.02.14.20023036

27. Frieden TR, Lee CT. Identifying and Interrupting Superspreading Events-Implications for Control of Severe Acute Respiratory Syndrome Coronavirus 2. *Emerg Infect Dis*. 2020. doi:10.3201/eid2606.200495

28. Rosenbaum L. Facing Covid-19 in Italy — Ethics, Logistics, and Therapeutics on the Epidemic’s Front Line. *N Engl J Med*. March 2020:NEJMp2005492. doi:10.1056/NEJMp2005492

29. Dichter JR, Kanter RK, Dries D, et al. System-Level Planning, Coordination, and Communication. *Chest*. 2014;146(4):e87S-e102S. doi:10.1378/chest.14-0738

30. Hick JL, Einav S, Hanfling D, et al. Surge Capacity Principles. *Chest*. 2014;146(4):e1S-e16S. doi:10.1378/chest.14-0733

31. King MA, Niven AS, Beninati W, et al. Evacuation of the ICU: Care of the critically ill and injured during pandemics and disasters: CHEST consensus statement. *Chest*. 2014. doi:10.1378/chest.14-0735

32. Biddison LD, Berkowitz KA, Courtney B, et al. Ethical Considerations. *Chest*. 2014;146(4):e145S-e155S. doi:10.1378/chest.14-0742
